# The effects of prone and supine positions on the regional distribution of ventilation in infants and children using electrical impedance tomography

**DOI:** 10.4102/sajp.v71i1.237

**Published:** 2015-05-29

**Authors:** Alison Lupton-Smith, Andrew Argent, Peter Rimensberger, Brenda Morrow

**Affiliations:** 1 School of Child and Adolescent Health, University of Cape Town, South Africa; 2 Paediatric Intensive Care Unit, Red Cross War Memorial Children's Hospital, South Africa; 3 Paediatric and Neonatal Intensive Care Unit, University Hospital of Geneva, Switzerland

## Abstract

**Background:**

Positioning of ill children is often used to optimise ventilation–perfusion matching, thereby improving oxygenation.

**Objectives:**

To determine the effects of supine and prone positions, and different head positions, on the distribution of ventilation in healthy, spontaneously breathing infants and children between the ages of 6 months and 9 years.

**Methods:**

Electrical impedance tomography measurements were recorded from participants in supine and prone positions. Head positions included the head turned to the left and right in supine and prone positions, and in the midline in the supine position. Distribution of ventilation was described using end-expiratory–end-inspiratory relative impedance change.

**Results:**

A total of 56 participants (boys = 31 [55%]; girls = 25 [45%]) were studied. The dorsal lung was significantly better ventilated than the ventral lung (*P* < 0.001) in both body positions. The majority of participants (83%) had greater ventilation in the dorsal lung in both positions, whilst five participants (10%) demonstrated consistently better ventilation in the non-dependent lung in both positions. Head position had no effect on the distribution of ventilation.

**Conclusions:**

This study demonstrates that the distribution of ventilation in healthy, spontaneously breathing infants and children in supine and prone positions is not as straightforward as previously thought, with no clear reversal of the adult pattern evident.

## Introduction

In ill and critically ill individuals, positioning is a non-invasive, inexpensive modality often used to improve oxygenation (Gillies, Wells & Bhandari 2012). Whilst many studies describe the effects of prone or supine positioning on factors such as mortality, arterial oxygenation, functional residual capacity, work of breathing and ventilation–perfusion matching (Curley *et al.* 2005; Gattinoni *et al.* 2001; Gillies *et al.* 2012; Pelosi, Brazzi & Gattinoni 2002), there are a limited number of studies that describe effects on the distribution of ventilation, particularly in infants and children older than 6 months.

To guide clinical practice, an understanding of what occurs under ‘normal’ conditions is essential. Until recently, positioning in the paediatric population has been based on the principle that children preferentially ventilate the non-dependent lung in side-lying positions, as suggested by earlier studies using invasive techniques (Davies *et al.* 1985; Heaf *et al.* 1983). This ‘paediatric pattern’ is the opposite of that observed in adults, who preferentially ventilate the dependent lung (West 2011). In adults, the dependent lung regions have a higher compliance, which contributes to the better ventilation in these regions. It was postulated that in infants and children, a greater degree of airway closure occurs in the dependent lung regions as a result of more positive pleural pressures (Davies *et al.* 1985; Heaf *et al.* 1983; Mansell, Bryan & Levison 1972; Papastamelos *et al.* 1995). Recent studies in neonates and infants up to 6 months of age have questioned this principle and suggested that the distribution of ventilation is towards the dependent lung (Frerichs *et al.* 2003; Hough 2008; Pham *et al.* 2011; Schibler *et al.* 2009), which is similar to what is seen in adults.

Lupton-Smith *et al.* (2013) recently reported the effect of side-lying positions on the distribution of ventilation in healthy, spontaneously breathing children older than 6 months. Response to positioning was found to be highly variable, with no clear ‘paediatric pattern’ of ventilation in either the right or the left lung. Only a few studies have reported the effects of both supine and prone positioning on ventilation distribution in both the ventral and dorsal lung regions in the paediatric population (Pham *et al.* 2011; Schibler *et al.* 2009). Although head position in supine and prone positions has been found to have an effect on the distribution of ventilation in neonates and newborns (Heinrich *et al.* 2006; Hough 2008), the effect in the older paediatric population is still unknown.

The current study aimed to determine the effects of supine and prone positions on the distribution of ventilation in healthy, spontaneously breathing infants and children using electrical impedance tomography (EIT). The effects of age and head position on the distribution of ventilation in prone and supine positions were also examined.

## Research design

### Research approach

This was a cross-sectional observational study, as described previously (Lupton-Smith *et al.* 2013).

### Research method

#### Population and sampling

A total of 56 healthy, spontaneously breathing, unsedated infants and children participated in the study. The participants were all between the ages of 6 months and 9 years and were recruited from out-patient clinics and day wards at Red Cross War Memorial Children's Hospital in Cape Town, South Africa. Participants were excluded if they had any respiratory disease (acute, chronic or symptoms in the preceding 6 weeks) or other factors, such as skeletal deformities, neuromuscular disease or cardiac conditions, impacting on respiratory mechanics. The same sample was used to describe the effect of side-lying on the distribution of ventilation (Lupton-Smith *et al.* 2013).

Ethical approval was obtained from the Human Research Ethics Committee of the University of Cape Town (ref 126/2012). Written informed consent was obtained from the parent or legal guardian of each participant and verbal or written assent was obtained from participants where age appropriate.

#### Procedure

The distribution of ventilation was measured using an EIT device (Goettingen Goe-MF II EIT System; Viasys/Carefusion, Germany). EIT is an emerging radiation-free, non-invasive imaging tool that is well validated and able to detect regional changes in ventilation in the lungs reliably and reproducibly (Bodenstein, David & Markstaller 2009; De Lema *et al.* 2008; Frerichs *et al.* 2007; Reifferscheid *et al.* 2011; Victorino *et al.* 2004).

Measurements were recorded in both supine and prone positions, with the head turned to the left and right in each, as well as with the head in midline in the supine position. Participants were first placed in the supine position with the head in midline. Subsequent head positioning was the participant's choice in both supine and prone positions. In both positions, participants lay flat, with their arms extended at their sides. Sixteen neonatal-sized electrodes (Blue Sensor BR-50-K, Ambu, Denmark), connected to the EIT device, were placed around the thorax at the level of the nipple line. EIT scans were generated at 13 scans per second. EIT measurements were recorded for as long as necessary (approximately one minute) to obtain a series of reproducible breaths in the required static position.

#### Offline analysis

Offline analysis was performed using Auspex Version 1.6 software (Viasys Healthcare, The Netherlands). The data were filtered to eliminate the effect of the heart (Zadehkoochak *et al.* 1992). A series of five consecutive reproducible breaths was selected for analysis. These were breaths of similar amplitude and without inspiratory or expiratory pauses.

**FIGURE 1:**
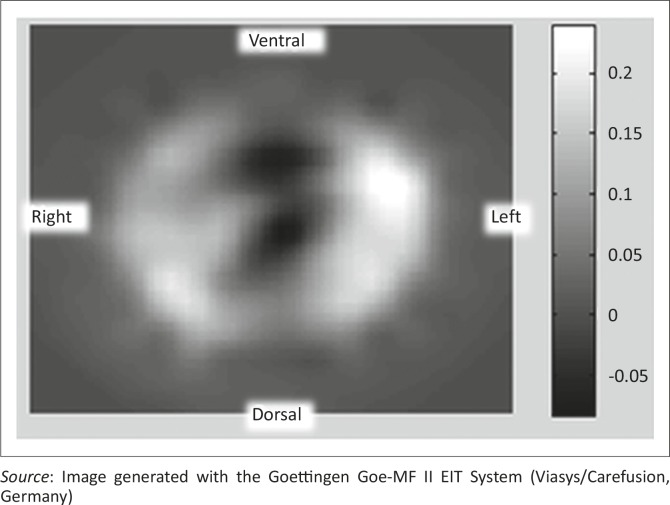
Functional electrical impedance tomography image from a participant (aged 4–6 years) in supine position. Lighter areas represent a higher impedance change (greater ventilation) and darker areas indicate areas of a smaller impedance change (poorer ventilation).

Functional EIT (fEIT) images, similar to the example shown in Figure 1, were generated using the average end-expiratory–end-inspiratory relative impedance change. Calculations were performed using the sum of pixel values of the fEIT images for both global and regional relative impedance change for specified regions of interest (ROIs). ROIs were determined as described by Pulletz *et al.* (2006), with 20% of the regression coefficient being used to determine the contour. ROIs examined were the ventral and dorsal lung regions, for analysis in supine and prone positions, and ventral, dorsal, left and right lung regions for the analysis of head position. Mean relative impedance change was used to compare ventral, dorsal and global ROIs in supine and prone positions, and the ventral, dorsal, left and right ROIs for the effect of head position.

To determine the ‘pattern’ of ventilation in both supine and prone positions, the proportion of ventilation (relative to global) was compared in the dependent and non-dependent ROI. A greater proportion of ventilation occurring in the non-dependent lung is described as a ‘paediatric pattern’, whilst a greater proportion of ventilation occurring in the dependent lung is described as an ‘adult pattern’. Where a greater proportion of ventilation was consistently distributed to the ventral or dorsal lung regions, these are further described as ‘dorsal’ or ‘ventral’ patterns, respectively.

The effect of prone and supine positions on ventilation distribution was determined with the head turned to the right in each of the positions.

#### Statistical analysis

Statistical analysis was performed using Statistica version 10 (StatSoft, Tulsa, USA). Data were tested for normality using the Shapiro-Wilk *W*-test and were found to be normally distributed. Multi-way analysis of variances (ANOVAs) were used to determine differences between age groups and ROIs in the different positions. Post hoc *t*-tests for dependent and independent variables were used to determine significant differences within and between groups and positions. Results are reported as means (± standard deviation) and 95% confidence interval. *P-*values ≤ 0.01 were considered statistically significant after applying the Bonferroni correction for multiple comparisons.

## Results

Sample characteristics are presented in Table 1. Incomplete measurements were obtained in both the supine and the prone positions for three subjects and in only the prone position for one subject. Results for global and regional ventilation in different positions are presented in Table 2. Global ventilation was similar in all supine and prone positions.

**TABLE 1:** Sample characteristics.

Age group	Number	Gender (Male:Female)	Mean age ± SD (years)	Respiratory rate ± SD (bpm)
6–12 months	10	6:4	0.9 ± 0.1	47.0 ± 7.4
1–3 years	18	11:7	2.8 ± 0.9	26.8 ± 5.3
4–6 years	18	7:11	5.6 ± 0.9	21.9 ± 3.6
7–9 years	10	7:3	8.7 ± 0.9	19.9 ± 2.9
**Total**	**56**	**31:25**	**-**	**-**

SD, standard deviation; bpm, breaths per minute.

**TABLE 2:** Mean relative impedance change (± standard deviation) as measured in different lung regions in prone and supine positions, including different head positions.

Lung region	SM (*n* = 56)	SL (*n* = 55)	SR (*n* = 56)	PL (*n* = 53)	PR (*n* = 52)
Left	16.8 ± 9.0	15.3 ± 7.1	16.3 ± 7.3	15.6 ± 6.8	16.7 ± 7.4
Right	17.6 ± 7.9	18.2 ± 6.6	16.8 ± 6.4	17.8 ± 7.1	17.6 ± 6.4
Ventral	14.5 ± 9.0†,‡	14.1 ± 7.2§	13.8 ± 6.8§	13.3 ± 6.4§	13.2 ± 6.2†,§
Dorsal	19.9 ± 8.2‡	19.5 ± 7.1	19.3 ± 7.3	20.1 ± 7.6	21.2 ± 7.6
Global	34.3 ± 16.2	33.6 ± 12.9	33.1 ± 12.9	33.4 ± 12.9	34.4 ± 12.8

PL, prone head to left; PR, prone head to right; SL, supine head to left; SM, supine head midline; SR, supine head to right.

†, *P* = 0.01 in the ventral lung between SM and PR; ‡, *P* = 0.001 between ventral and dorsal lung regions in SM; §, *P* < 0.001 between ventral and dorsal lung regions in SL, SR, PL, PR.

### Ventral and dorsal lung regions

There was no statistical difference in ventilation within the ventral or dorsal lung regions between supine and prone positions (Table 2). However, the dorsal lung region was significantly better ventilated than the ventral lung region in both supine and prone positions (*P* ≤ 0.001).

### Patterns followed

The majority of the participants (*n* = 43; 83%) consistently showed greater ventilation in the dorsal lung in both supine and prone positions, whilst two participants (4%) demonstrated greater ventilation in the ventral lung in both supine and prone positions. Five participants (10%) consistently demonstrated the paediatric pattern in both positions, whilst two (4%) consistently demonstrated the adult pattern in both positions (Table 3).

**TABLE 3:** Number of participants following the paediatric pattern or adult pattern in the supine or prone positions, together with a summary of the overall pattern in the two positions.

Age group	Supine		Prone		Overall^c^			
	Paed^a^	Adult^b^	Paed	Adult	Paed	Adult	Ventral	Dorsal
6–12 months	1	6	6	1	1	1	0	5
1–3 years	4	13	16	1	3	0	1	13
4–6 years	1	17	16	2	1	1	0	16
7–9 years	1	9	9	1	0	0	1	9
**Total**	**7**	**45**	**47**	**5**	**5**	**2**	**2**	**43**

^a^Paediatric pattern refers to greater ventilation in the non-dependent lung.

^b^Adult pattern refers to greater ventilation in the dependent lung.

^c^Overall pattern refers to the pattern followed consistently in both supine and prone positions

Paed, paediatric pattern.

### The effect of head position

There was no significant effect of head position on the distribution of ventilation within individual lung regions and between left and right, and ventral and dorsal lung regions, respectively. Global ventilation was not affected by different head positions in either supine or prone positions (Table 2).

### The effect of age

There were no age-related differences in mean relative impedance change in ventral (*P* = 0.4) or dorsal lung regions (*P* = 0.7), nor globally (*P* = 0.8), due to body position. The effect of head position was not statistically different amongst age groups in any region in either body position.

## Discussion

This study examined the distribution of ventilation in healthy, spontaneously breathing infants and children in supine and prone positions, with different head positions.

Whilst mean relative impedance change results suggest that the dorsal lung was better ventilated in both supine and prone positions, analysis of the pattern of ventilation showed that this was not the case in all participants. The previously described pattern of ventilation in the paediatric population (Davies *et al.* 1985; Heaf *et al.* 1983) is not supported by the results of this study, with ventilation distribution in the majority of participants (87%) remaining unaffected by supine or prone positioning.

A recent study in neonates (Hough *et al.* 2012) reported that ventilation in the dorsal lung regions was significantly higher than in the ventral lung regions in both supine and prone positions, in keeping with our results. Similar results for the supine position were found by Pham *et al.* (2011), who reported an increase in ventilation in the dependent (dorsal) lung in infants at 2 weeks, 3 months and 6 months of age. These findings are in line with our results in the older paediatric population; however, whether this ‘pattern’ also occurred in the prone position in infants under 6 months was not examined in the previous study (Pham *et al.* 2011). In another study, neonates were reported to show an equal ventilation distribution between dependent and non-dependent lung regions in both supine and prone positions (Schibler *et al.* 2009). In that study, measurements were taken during non-rapid eye movement sleep, during which the breathing pattern is more regular. In the present study, however, measurements were taken in awake infants and children, with an irregular breathing pattern to be expected. These differences in study conditions may account, at least in part, for the difference in results.

The distribution of ventilation in different body positions (towards the dependent lung) in the adult population has been well established and is thought to be primarily as a result of gravitational forces on the lungs (Bryan, Milic-Emili & Pengelly 1966; Gattinoni *et al.* 1991; Kaneko *et al.* 1966). Pleural pressures become increasingly more positive down the vertical axis through which gravity acts (West 2011). Consequently, the dependent lung portions have lower resting volumes and are, therefore, able to expand more during inspiration than the non-dependent portions (Frerichs, Hahn & Hellige 1996; Kaneko *et al.* 1966; Milic-Emili *et al.* 1966). Infants and children, however, have higher closing volumes and a lower functional residual capacity as a result of a more compliant chest wall and 'stiffer’ lungs (Cooper, Mellins & Mansell 1981; Davies *et al.* 1985; Hatch & Fletcher 1992; Heaf *et al.* 1983), resulting in preferential ventilation in the non-dependent lung. The disparity found between adults and children in previous studies examining ventilation distribution was explained by these differences in the respiratory mechanics as a result of a maturing respiratory system (Davies, Helms & Gordon 1992; Hatch & Fletcher 1992). Our finding, namely that there is greater ventilation in the dependent lung in the supine position, can be explained according to the effect of gravity on the distribution of ventilation seen in the adult population. In the prone position, however, it has been shown that there is a more homogenous distribution of lung tissue and alveolar inflation, as well as a smaller pleural pressure gradient, which results in a more uniform distribution of ventilation (Pelosi *et al.* 2002). This may account for the lack of gravity-dependent ventilation distribution seen in the prone position. Bryan *et al.* (1966) suggested that the distribution of ventilation depends on the magnitude of the gravitational forces acting on the lungs. As the vertical axis through which gravity acts is reduced in horizontal postures, the effect on the lung tissue may be less and may account for the similarities between supine and prone positions.

Whilst the majority of participants demonstrated a similar pattern of distribution, some participants demonstrated either a consistent adult or paediatric pattern. Variations in breathing pattern may account for the different patterns seen. It has been established in the adult population that breathing at lower lung volumes results in greater ventilation in the non-dependent lung regions (Milic-Emili *et al.* 1966; Schnidrig *et al.* 2013). Hutten *et al.* (2008) found that activity of the respiratory muscles varied with each breath in neonates, which may save energy whilst maintaining optimal ventilation. This fluctuating use of respiratory muscles may account for the variability in the distribution of ventilation, although further investigation is needed to test this hypothesis.

Unlike the findings of Heinrich *et al.* (2006), who reported significant effects of head position on the distribution of ventilation in the right lung region in a cohort of neonates, head position had no effect on the distribution of ventilation in participants (from an older paediatric population) in our study.

Lung perfusion may account for some of the impedance changes detected, as lung perfusion is affected by gravity. However, these changes are reported to account for less than 5% of the recorded impedance change (Frerichs *et al.* 2009).

The previously described ‘paediatric pattern’ of ventilation was not consistently observed in the present study, with the majority of participants showing consistently greater ventilation in the dorsal lung region. In clinical practice, therefore, it cannot be assumed that every infant or child preferentially ventilates the non-dependent lung. Rather, it is suggested that the selection of the position used clinically should be guided by the individual's response and preference. This response could be determined by monitoring parameters such as heart rate, respiratory rate and pattern, oxygen saturation and auscultation. Clinical use of EIT may also be valuable in determining real-time effects of positioning.

Whilst this study reveals that the distribution of ventilation in infants and children is more complex than previously thought, there are several limitations. It is recognised that ventilation distribution is affected by end-expiratory and tidal volumes (Schnidrig *et al.* 2013); direct measures of these may have strengthened this study. The order of positions was not randomised owing to cooperation challenges. We were, therefore, unable to determine whether the order of positioning had any effect on the distribution of ventilation. The position was difficult to standardise, given that the participants were healthy and unsedated. Lastly, participants spent only a short amount of time in each position; therefore, we were unable to determine whether distribution of ventilation is time dependent.

Despite these limitations, this study provides novel insights regarding the distribution of ventilation in healthy, spontaneously breathing infants and children in response to supine and prone positioning. In addition, it is the first to examine the effects of head position on the distribution of ventilation in an older paediatric population. This study provides baseline data against which future clinical studies can be compared. Further research is warranted to determine the effects of mechanical ventilation and respiratory disease on ventilation distribution, the effect of time spent in a specific position on ventilation distribution, and, specifically, the effect of prone turning in mechanically ventilated children with acute respiratory distress syndrome.
